# Assessment of surgery residents' knowledge of medical ethics and law. Implications for training and education

**DOI:** 10.25122/jml-2022-0035

**Published:** 2023-03

**Authors:** Shabnam Bazmi, Parsa Kiani, Seyed Ali Enjoo, Mehrzad Kiani, Elham Bazmi

**Affiliations:** 1 Medical Ethics Department, School of Traditional Medicine, Shahid Beheshti University of Medical Sciences, Tehran, Iran; 2 Simon Fraiser University, Vancouver, Canada; 3 Iranian Legal Medicine Organization, Legal Medicine Organization Research Center, Tehran, Iran

**Keywords:** ethics, law, education, surgery resident, knowledge

## Abstract

Medical ethics and law are essential topics that should be included in medical residency programs. However, surgery training programs in Iran lack a specific course in medical ethics and law, which can lead to patient dissatisfaction with surgical outcomes. This study aimed to assess surgery residents' knowledge of medical ethics and law and suggest improvements for future residency programs. This descriptive cross-sectional study involved 112 surgery residents from six teaching hospitals. A valid and reliable questionnaire comprising 15 items on medical ethics and 12 items on medical law was used to assess participants' knowledge. Most participants were female (31-40 years old), and their mean knowledge score for medical ethics was 3.26±0.53 out of 5, with the lowest score in "futile treatment and DNR orders." The mean knowledge score for medical law was 3.69±0.69, with the lowest score in "surrogate decision-maker." Age did not affect residents' knowledge, but gender did, with female residents demonstrating significantly better knowledge of medical ethics (3.344/5 vs. 3.112/5) and law (3.789/5 vs. 3.519/5). Surgery residents had a relatively favorable knowledge of medical ethics and law, but they require further training in some areas to improve their knowledge. Training should include journal clubs, role-play programs, standardized patient programs, and debates to achieve better results, as purely didactic lectures appear inadequate.

## INTRODUCTION

Medical ethics has become an essential topic in residency programs, particularly in surgery residencies, as surgeons encounter ethical issues more frequently than many other clinicians [1]. The method used to teach medical ethics is critical in medical training, and empowering medical residents to communicate bad news, disclose medical errors, and discuss other clinical-ethical considerations is a major part of these teaching programs [2]. However, some surgery residents believe that their role in the hospital is not sufficiently clear to patients, and despite their commitment to disclosing errors and communicating effectively, acting correctly is not always easy for them [3]. Patients also have a limited understanding of residents' responsibilities in hospitals [4]. Surgeons experience moral distress when making ethical decisions, such as uncertainty about beginning or terminating treatments, allocating scarce resources, following laws and regulations, and dealing with some of their colleagues’ poor performance [5]. However, the professional and ethical abilities of surgery residents do not necessarily improve during residency programs, and a lack of standard academic admission requirements is another problem in this area [6].

Residency training programs should include leadership training as well as technical and clinical learning [7]. Due to the general shift toward more outpatient care, some ethical knowledge and skills may be diminished in surgery residents, and the ever-changing environment of a teaching hospital makes the management of the ethical climate difficult [8].

Improving the knowledge of ethics and law among different medical professional groups, including physicians, nurses, residents, and interns, could diminish ethical problems by improving professional communications, protecting patients' rights, especially vulnerable ones, and reducing errors. Guntik showed that it is essential to emphasize ethical issues in trials and surgical interventions and focus on professionalism in surgery residency programs [9]. Helft offered evidence suggesting that the bulk of ethical knowledge of surgery residents pertains to their undergraduate studies, and they had received limited ethical content during their residency. Posing the question of how and how much ethical issues can be integrated into residency training, Helft further argued that focusing on surgery residents and their common moral dilemmas can be beneficial [10]. Our medical ethics curriculum should cover ethical problem-solving and decrease students' and residents' emotional discomfort when encountering ethical dilemmas [11].

The current method of teaching medical ethics to surgery residents needs to change, and identifying the current knowledge of the target group is the first step toward improvement [12]. Despite various studies conducted worldwide, there have been few studies on surgery residents in Iran. Therefore, the present study aims to evaluate Iranian surgery residents' knowledge of essential topics in medical ethics and law, which should have been included in their training programs.

## MATERIAL AND METHODS

This descriptive, analytical, cross-sectional study included general surgery residents in six major teaching hospitals in the capital city of a developing country (Tehran/Iran). Stratified random sampling was employed to select the participants from all six hospitals affiliated with the university, and 160 residents were finally selected. Initially, a biostatistician was consulted and a pilot study was conducted on 20 surgery residents. The Epi Info™ App (version 16, released November 2018) was used to calculate the sample size, considering the following parameters: population size =9820 (unique visitors of the website at that moment), 95% confidence interval, and level of precision of estimate within 5% of either side of the true population proportion. Accordingly, a minimum of 110 cases were required for the study. To account for missing data, as previously mentioned, 160 surgery residents were selected.

The participants were briefed on the questionnaires, and their informed consent for participation was obtained. The researcher-designed questionnaire had three parts: demographic data, clinical ethics knowledge (Appendix 1), and medical law information (Appendix 2).

Appendices

The essential topics in medical ethics and law were determined as per the national board of surgery, the national board of medical ethics, syllabi of medical ethics, and medical law content in the curricula of relevant fields, as approved by the Vice Chancellor of Education of the Ministry of Health and Medical Education of Iran and the Iranian Medical Council's Codes of Professional Conduct. All four faculty members of the Medical Ethics Department of Shahid Beheshti University of Medical Sciences approved the trustworthiness of the data collection form (two males and two females; one full professor, two associate professors, and one assistant professor). The validity of the form was measured according to the four experts’ opinions, and the Content Validity Index (CVI) was calculated as 90%. In addition, the internal consistency and reliability were determined at 0.8 using Cronbach’s alpha. To get the forms filled out, the researchers visited the selected hospitals and asked all the intended residents to complete the questionnaire if they consented to participate in the research. Necessary information was disclosed to the participants to help them decide, together with detailed explanations about anonymity and confidentiality of data. Those willing to participate in the research were then asked to complete the forms.

The data collection form consisted of two demographic items and 27 (15+12) other items covering the knowledge parts. The clinical ethics knowledge part of the instrument consisted of 15 items on the following issues: medical ethics and the professional self-regulation of doctors, paternalism and the doctor-patient relationship, the meaning of the principle of respect for patient autonomy, the professional confidentiality rule and its consideration as an absolute or a relative rule, consent of the brain-dead donor’s for organ transplantation and the concept of presumed consent in such cases, the living will of the deceased donor, informed consent for pediatric surgery and the proper surrogate decision-makers and the child’s assent, the ethical acceptability of financial incentives for transplant organ donation, the concept of euthanasia and its passive type, visitors requesting to not resuscitate their patient in the case of cardiopulmonary arrest because of the patient’s severe pain and poor economic status, the ethics of research on human participants and their right to withdraw from participation at any part of the research, the matter of prisoners’ participation in research and the concept of vulnerable people, therapeutic misconceptions, i.e., participants of medical research assuming that the study offers them better individual care services, the quantitative definition of futile treatment, and considering negotiation with the patient as the best approach when facing futile requests on their part (Appendix 1).

The medical law information part of the instrument consisted of 12 items on the following subjects: the penalty for breaching medical confidentiality in the country’s national law and the best approach to resolve such dilemmas, the effect of consent and bara’at (a concept overlapping informed consent in the Shia jurisdiction mirrored in the IR Iran law) in the case of medical lawsuits, the patient’s right to know their surgeon and their residents/aids, negligence medical errors, and the law, approaching the malpractice of peers, improper legal surrogate decision-maker and the incompetent patient, requirements for a good informed consent, legal issues of consent in full emergency cases, the concept of the patient’s capacity to fill out the consent form, and the influence of truth-telling on the frequency and decline of lawsuits. The items in this part of the instrument contained three different medical scenarios with difficult legal considerations. The goal of the study was to evaluate the knowledge and information of surgery residents on topics in medical ethics and law based on the proposed questionnaire items (Appendix 2), which were scored on a 5-point Likert scale (5 for the correct answer and 1 for the least correct answer).

A score of 1-1.99 was considered unfavorable, 2-2.99 relatively unfavorable, 3-3.99 relatively favorable, and 4-5 favorable.

To calculate CVI (Content Validity Index), the questionnaire was given to four experts (two males and two females; one full professor, two associate professors, and one assistant professor), and they were asked to comment on the relevance, clarity, and simplicity of the 15 items on clinical ethics knowledge and the 12 items on medical law information based on a four-point Likert scale (1: unfavorable, 2: relatively unfavorable, 3: relatively favorable 4: favorable). For this purpose, CVI was measured using the number of experts rating 3 and 4 for each item, divided by the total number of experts [13]. Hyrkas et al. (2003) recommended a score of 0.79 and above for accepting the items based on their CVI [14]. In this study, the CVI of both parts of the form was reported as 0.90. Interclass correlations (ICC) were also calculated in 112 participants after 30 days to evaluate the temporal stability. To determine the internal consistency, Cronbach’s alpha coefficient was calculated, reported as 0.80 for both parts of the form.

The data were analyzed using descriptive statistics, including frequency distributions, means, and standard deviations, and inferential statistics, such as Pearson’s correlation coefficient, independent t-test, Chi-square test, and ANOVA. The Kolmogorov-Smirnov test was used to evaluate the normal distribution of the data. SPSS 22 was used for all the tests.

## RESULTS

Out of the 160 residents, 112 participated in the study (response rate =0.7). Some demographic characteristics of the participants are presented in Table 1. Most respondents were between the ages of 31-40 (n=71, 63.4%) and had graduated more than five years before the study as general practitioners (n=61, 54.5%). While most of the residents (70 (62.5%) and 90 (80.4%), respectively) believed that lectures, conferences, and workshops on medical ethics and law were effective and necessary, 83 (74.1%) reported not having taken any training course in ethics or law after obtaining their MD certificate and before beginning the residency.

**Table 1:** Demographic characteristics of participants.

Characteristics	Number (%)
Sex	
**Male**	40 (35.7%)
**Female**	72 (64.3%)
Age	
**20–30**	34 (30.4%)
**31–40**	71 (64.3%)
**≥41**	7 (6.3%)
Years after graduation	
**<5**	61 (54.5%)
**6–7**	21 (18.8%)
**8–10**	10 (8.9%)
**>10**	20 (17.9%)
Residency year	
**First**	35 (31.3%)
**Second**	32 (28.6%)
**Third**	26 (23.2%)
**Fourth**	19 (17.0%)
Having a course of ethics or law education course	
**Yes**	28 (25%)
**No**	84 (75%)
Declared effectiveness	
**Yes**	70 (62.5%)
**No**	42 (37.5%)
Declared necessary	
**Yes**	90 (80.4%)
No	22 (19.6%)

Table 2 presents the frequency distribution of medical ethics and law knowledge based on the scores obtained by the surgery residents. The ‘relatively favorable’ scores ranged from 1.99 to 2.99 and were the most frequent in both ethical knowledge and law information.

**Table 2:** Frequency of clinical ethics knowledge and medical law information dimension among participants.

	Unfavorable N (%)	Relatively unfavorable N(%)	Relatively favorable N (%)	Favorable N (%)
Clinical ethics knowledge	-	33 (29.5%)	69 (61.6%)	10 (8.9%)
Medical law information	2 (1.8%)	13 (11.6%)	50 (44.6%)	47 (42%)

The t-test revealed statistically significant relationships between clinical ethics knowledge and medical law information with gender (P=0.026 and P=0.046, respectively). In addition, the results represented a direct relationship between the score obtained by the residents and whether they had taken a course on medical ethics and law (P=0.004 and P=0.006, respectively). Table 3 compares the mean scores of the participants in each domain of clinical ethics knowledge and medical law information based on their demographic characteristics.

**Table 3:** Comparing the mean scores per clinical ethics knowledge and medical law information dimension (according to the demographic characteristics of participants).

Demographic characteristics	Clinical ethics knowledge Mean score (SD)	Medical law information Mean score (SD)
Sex		
**Male**	3.11 (0.46)	3.51 (0.76)
**Female**	3.34 (0.55)	3.78 (0.63)
**P-value**	0.026	0.046
Age		
**20–30**	3.25 (0.48)	3.60 (0.7)
**31–40**	3.26 (0.560	3.73 (0.69)
**≥41**	3.21 (0.49)	3.64 (0.58)
**P-value**	0.97	0.64
Years after graduation		
**<5**	3.21 (0.51)	3.63 (0.66)
**6–7**	3.41 (0.58)	3.85 (0.78)
**8–10**	3.14 (0.41)	3.46 (0.88)
**>10**	3.29 (0.5)	3.81 (0.53)
**P-value**	0.41	0.35
Residency year		
**First**	3.25 (0.56)	3.64 (0.75)
**Second**	3.21 (0.54)	3.52 (0.74)
**Third**	3.37 (0.52)	3.87 (0.61)
**Fourth**	3.18 (0.48)	
**P-value**	0.62	0.22
Having a course of ethics or law education course		
**Yes**	3.02 (0.55)	3.39 (0.633)
**No**	3.34 (0.50)	3.89 (0.78)
**P-value**	0.004	0.006
Declared effectiveness		
**Yes**	3.35 (0.56)	3.78 (0.43)
**No**	3.1 (0.43)	3.54 (0.76)
**P-value**	0.065	0.085
Declared necessary		
**Yes**	3.27 (0.54)	3.73 (0.67)
**No**	3.19 (0.49)	3.50 (0.72)
**P-value**	0.51	0.16

## DISCUSSION

The present study evaluated the knowledge and understanding of surgery residents at Shahid Beheshti University of Medical Sciences in Tehran, Iran, regarding medical ethics and law. The results revealed that the mean score in the “clinical ethics knowledge” dimension was 3.26 among participants (range:3-3.99), and this dimension thus had a relatively favorable status among surgery residents. Despite the relatively favorable scores in this dimension, the following factors received a score between 1.99 and 2.99 and were not favorable: patient autonomy (No. 3), gift rewarded to organ donor (No. 7), visitors requesting not to resuscitate their patient (No. 10), withdrawal of participants from the research without any explanations (No. 11) and performing research on prisoners (No. 12).

The mean score in the dimension of “medical law information” was 3.3 among participants, which falls between 3 and 3.99, and this dimension thus had a relatively favorable status in the surgery residents. Only one item, i.e., proper surrogate decision-maker (No. 7), was relatively unfavorable.

It can therefore be argued that “medical law information” and “clinical ethics knowledge” were generally favorable in surgery residents at Shahid Beheshti University of Medical Sciences in Tehran, Iran. However, their “medical law information” status was slightly better. Physicians and surgeons often pay more attention to the legal aspects of ethical issues. Greater educational efforts are required in some areas of ethics (autonomy, gift rewarded to the organ donor, withdrawal of participants from the research, and research on vulnerable participants) and law (legal surrogate decision-makers and informed consent).

This study found that age was not a significant factor in the knowledge of ethics and law among surgery residents. However, gender appeared to have an impact, with female residents demonstrating significantly greater knowledge in ethics (3.344/5 vs. 3.112/5) and law (3.789/5 vs. 3.519/5) compared to their male counterparts.

In a study on internal medicine residents, Rosenbaum et al. showed that only 10% of respondents had not been involved in an ethically challenging scenario directly. They reported challenges related to truth-telling, respecting patients’ preferences, harm prevention, and proper surrogate decision-makers when patients lack decision-making capacity. Their findings correspond to our study regarding respect for the patient’s autonomy and making the best decisions for patients lacking decision-making capacity. However, the two studies differ in their approach; Rosenbaum's study explored experiences through in-depth interviews, while the present study scored surgery residents' knowledge and approaches to clinical-ethical challenges. Rosenbaum et al. did not find a difference between the participants in terms of gender or year of residency, while the present study showed that female residents had significantly more knowledge on ethical and legal issues, which could be due to differences in the methodologies of the two studies [15].

The study by Howard et al. identified important topics to be covered in medical ethics classes for surgery residents, including conflicts of interest, doctor-patient relationships, end-of-life care, innovation and research, religious and cultural issues, resource allocation, surgical competence, and truth-telling. Their study also found that residents faced challenges in obtaining consent, making disclosures, providing end-of-life care, maintaining surgical competence, and making decisions. The authors noted that surgical residents are often evaluated based on their technical rather than ethical skills. Some residents reported that although their formal curriculum covered some of these topics, they felt the lessons were insufficient [16].

Martinez et al. and Lynch similarly believed that medical errors and communication are some of the most problematic ethical-legal issues in surgery residency. However, the present study found that participants’ knowledge in these areas was better than expected, although this may reflect a gap between knowledge and practice [17-19]. Similar findings have been reported in other studies conducted in Iran [20].

While some authors have discussed interpersonal problems in clinical practice or the abuse of medical students as medical ethics problems, these issues actually fall under general or organizational ethics in healthcare [21].

Monrouxe et al. identified patient safety, dignity, and healthcare worker abuse in the workplace as the most problematic ethical issues for healthcare students. As mentioned above, although general ethical problems in the workplace are very important, they may not necessarily be considered clinical ethical issues. Similar to Monrouxe et al., we found that ensuring patients' well-being and respecting their dignity are bioethical issues. In our study, dignity, and autonomy were not as favorable as in Monrouxe’s study. In contrast to the cited study, the present study found no problems in the surgery residents’ knowledge of patient safety and medical errors, possibly due to the gap between theory and practice [21].

This study has several strengths, including that the researchers were all physicians taking postgraduate courses in medical ethics, law, and forensic medicine. Furthermore, they were responsible for administering ethics education at the university, which enabled them to use the study results to develop the medical students' and residents' curriculum within the guidelines of the Ministry of Health and Medical Education. The findings of the study can contribute to a mutual understanding and common discourse between medical ethics and surgery professors and can provide a path for the joint development of surgery ethics education and practice. After completing the study, teaching programs and workshops were designed and implemented for residents in the plastic surgery, general surgery, and internal medicine wards of the teaching hospitals. Additionally, the residents’ ethics knowledge and ethical behaviors in all areas are now part of their annual promotion exam. Future studies are recommended to further assess these needs and plan appropriate interventions for improving medical ethics education among residents. Additionally, one of the limitations of the study was the difficulty in coordinating and persuading busy surgery residents to participate in the research, despite efforts to minimize the number of questions asked.

## CONCLUSIONS

The surgery residents in this study demonstrated lower than acceptable knowledge on some particular ethical issues, including patient autonomy, rewarding gifts to organ donors, futile treatments, research ethics, and vulnerable participants. However, they showed an overall acceptable to favorable level in other areas. Further training and focus on these subjects are therefore required. In terms of legal considerations, the only area in which the residents’ knowledge was less than acceptable pertained to the issue of proper legal surrogate decision-makers for patients lacking decision-making capacity in order to obtain informed consent; they thus require further training on this particular subject. The authors also suggest implementing educational plans for patients in surgery wards to improve their familiarity with the surgical team members.
