# Supplementary material for: Assessment of surgery residents' knowledge of medical ethics and law. Implications for training and education
Source: J Med Life. 2023 Mar;16(3):406–11. doi: 10.25122/jml-2022-0035 (PMC10165531; doi:10.25122/jml-2022-0035)
Supplement: Supplementary file 1 — Appendices [file JMedLife-16-406-s001.pdf]

Appendices

Appendix 1. Clinical ethics knowledge dimension.

Statements	Suggested mean=3			t-test indexes		
	Number	Mean	Standard deviation	T	Degree of freedom	Sig.
Medical ethics reflects the way for regulating behavior and shows the principles governing this.	112	4.0625	1.18763	9.468	111	.000
Paternalism in medicine means pure authority of the physician.	112	3.2411	1.24658	2.047	111	.043
Autonomy in medicine means not a physician's clinical diagnostic skills.	112	2.4464	1.33450	-4.390	111	.000
Confidentiality in medicine is not absolute, but the secrets of patients should not be revealed.	112	3.7321	1.34219	5.773	111	.000
In organ transplant from the corps, presumed consent means that the deceased person is satisfied with the donation unless he or she has insisted or requested otherwise during his or her lifetime.	112	3.6964	1.24365	5.926	111	.000
In surgery for children, parental consent and the child's cooperation and assent are required.	112	4.0893	1.11140	10.372	111	.000
Receipt of rewards for organ donation (rewarded gift) by the organ donor is morally acceptable.	112	2.8482	1.30287	-1.233	111	.220
Euthanasia means ending the life of a human being who can survive.	112	3.5268	1.36863	4.073	111	.000
Passive euthanasia means preventing action that can prolong a person's life.	112	3.8571	1.16910	7.759	111	.000
The patient is in severe pain and incurs high medical costs. Her daughter is therefore seeking discontinuation of medical treatment and DNR. This request is acceptable despite the patient's condition.	112	2.3482	1.30632	-5.280	111	.000
According to the executive regulations of ethical principles in the research of medical sciences in the country, the participant (research subject) can leave the research whenever he wants	112	2.5179	1.40130	-3.641	111	.000
According to the executive regulations of ethical principles in medical research in our country, the participation of prisoners in medical research is not prohibited.	112	2.8036	1.22907	-1.691	111	.094
The term "therapeutic misconception" in medical research means that patients generally believe that research methods are meant to increase their individual care.	112	3.4107	1.03588	4.196	111	.000
Qualitative definition of futile treatment is that "physicians, based on personal and shared experience with colleagues or considering empirical information reported, conclude that in the last 100 cases, the intended treatment has been ineffective".	112	3.1607	1.07848	1.577	111	.118
The best way to deal with inappropriate patient requests is to communicate openly and discuss the problem and its solution.	112	2.5268	1.30805	-3.829	111	.000
Total	112	3.2613	.53432	5.176	111	.000

Appendix 2. Medical law information dimension.

Statements	Suggested mean=3			t-test indexes		
	Number	Mean	Standard deviation	t	Degree of freedom	Sig.
The best person to deal with about confidentiality is the patient.	112	4.1607	1.16676	10.528	111	.000
Punishment for disclosing the secrets of patients in the current laws of Iran is a fine of three months and one day of imprisonment or payment of 150 to 600 thousand Tomans.	112	3.5000	1.03975	5.089	111	.000

Appendix 2. Continued.

Statements	Suggested mean=3			t-test indexes		
	Number	Mean	Standard deviation	t	Degree of freedom	Sig.
According to Iranian law, in addition to consent, the patient must be acquitted(baraat), even if scientific and legal standards are observed.	112	3.7500	1.16634	6.805	111	.000
The patient has the right to know the surgeon and the assistants accompanying him/her in the surgery.	112	4.1250	1.10792	10.746	111	.000
During surgery, surgical instruments are left in the patient's abdomen. In this case, the surgeon has committed medical negligence.	112	3.6339	1.36227	4.925	111	.000
A person has been referred to the emergency department of a training hospital and complains of lower abdominal pain and anorexia. The assistant surgeon on duty prescribes painkillers without further diagnostic and paraclinical examinations. After a few hours, the patient goes to another center with the same symptoms as the pain worsens, and clinical examinations and diagnostic and paraclinical procedures show that he has acute appendicitis. The action of the Assistant Surgeon of Surgery in the absence of a thorough examination at the initial medical examination is a kind of negligence.	112	3.5982	1.35207	4.682	111	.000
The 17-year-old was taken to the emergency room at 11 a.m. with his uncle, a retired police officer, in pain with the diagnosis of an inguinal hernia, and he needs an operation tomorrow morning. Can his uncle give consent for this surgery?	112	2.6429	1.41330	-2.674	111	.009
Three components are required to obtain valid patient consent: adequate information, decision-making capacity, and voluntariness	112	3.9911	1.11900	9.373	111	.000
It is not necessary to seek consent during an emergency (a condition that requires immediate treatment to save a patient's life or health).	112	3.8750	1.26722	7.307	111	.000
Patients' capacity to consent means that they can understand the information related to their decision and the predictable side effects of it.	112	4.0446	1.12617	9.817	111	.000
Truthtelling reduces patients' protest against doctors.	112	3.7857	1.15804	7.180	111	.000
According to international medical ethics guidelines, disclosure of patients' secrets is not ethical under any circumstances.	112	3.2054	1.38940	1.564		.121
**Total**	112	3.6927	.69018	10.622	111	.000
